# Supplementary figures and images for: Indoor Inactivation of SARS-CoV-2 Virus by Liquid Hyperoxygen
Source: Pathogens. 2024 Mar 11;13(3):244. doi: 10.3390/pathogens13030244 (PMC10974922; doi:10.3390/pathogens13030244)

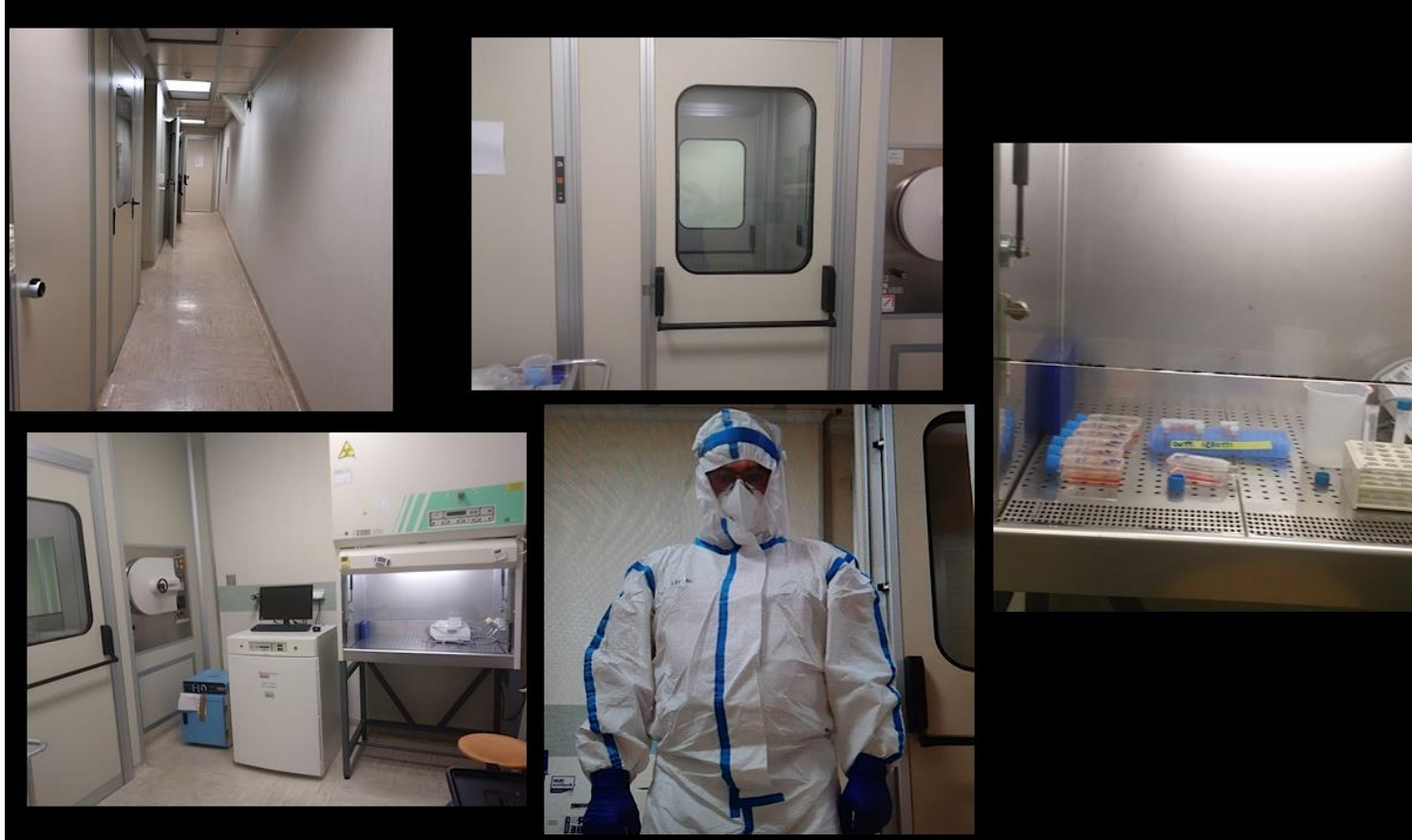

**Figure S1.** Demonstration of the working space.

Supplement: Supplementary file 1 [file pathogens-13-00244-s001.zip › pathogens-2844626-supplementary.pdf]
